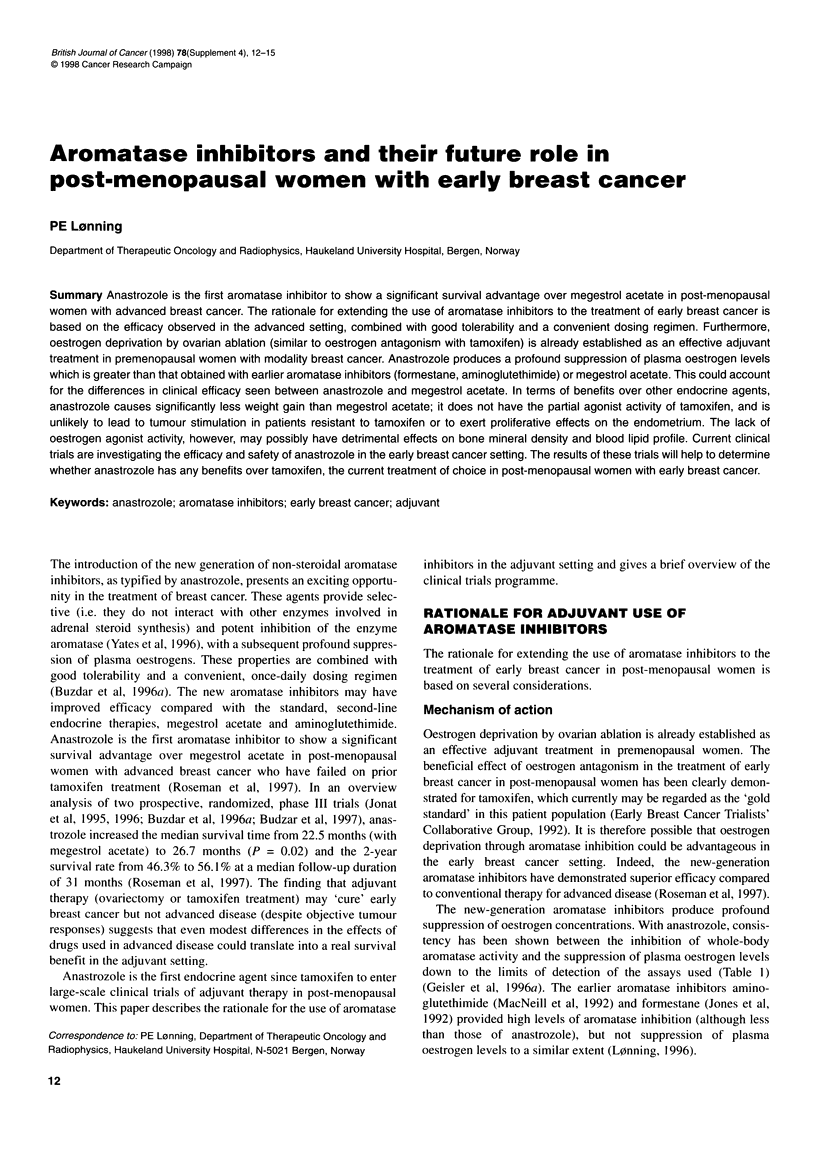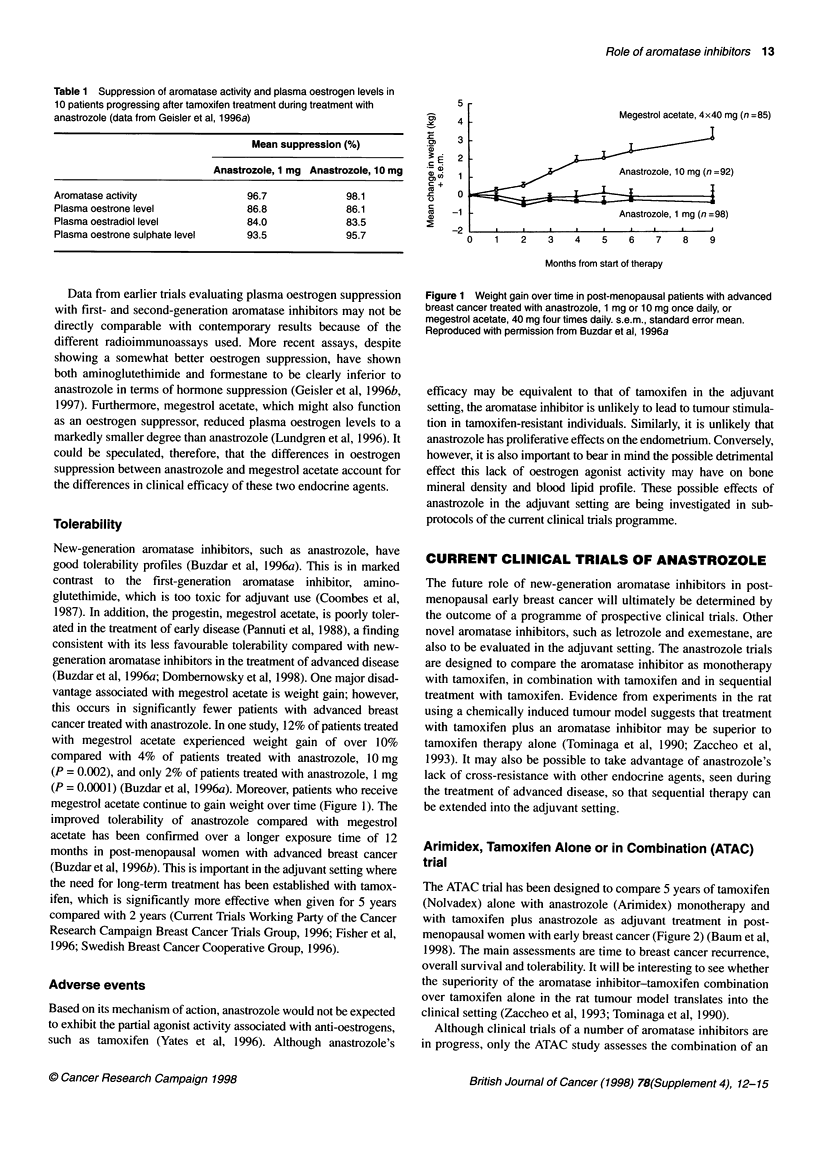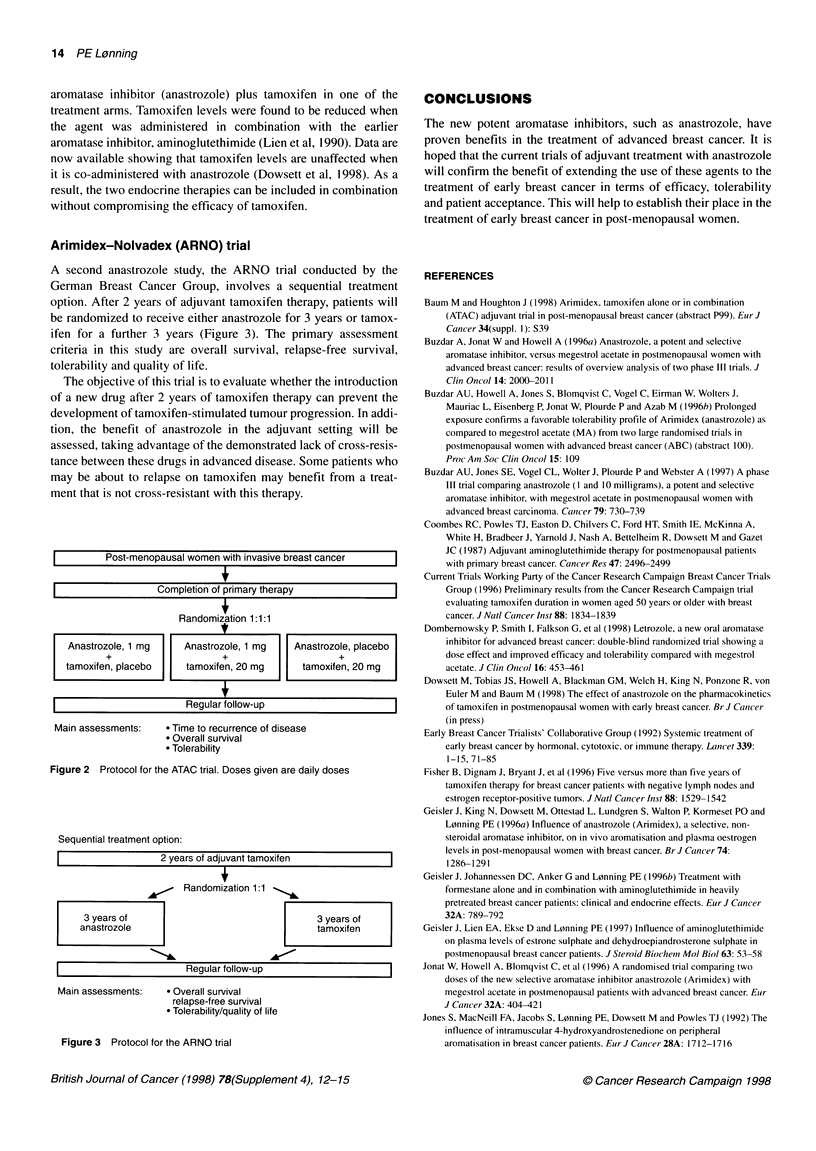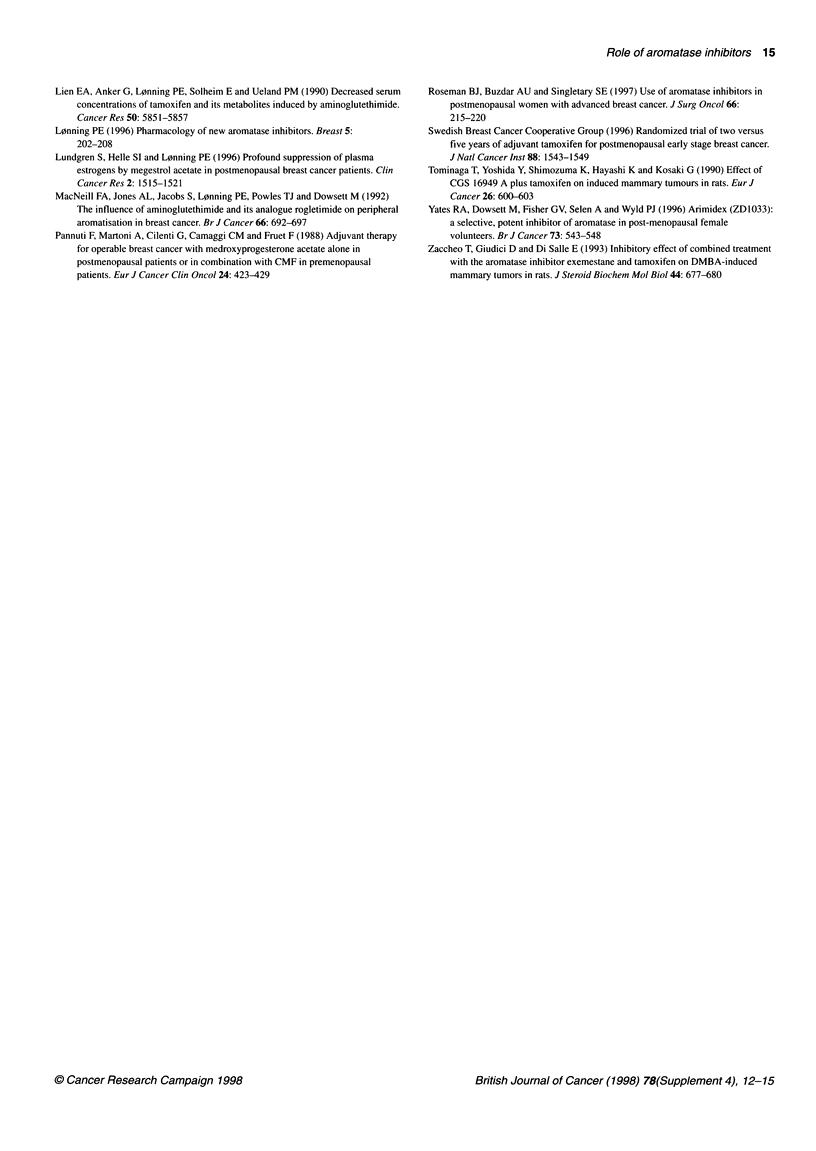# Aromatase inhibitors and their future role in post-menopausal women with early breast cancer.

**DOI:** 10.1038/bjc.1998.756

**Published:** 1998-09

**Authors:** P. E. Lønning

**Affiliations:** Department of Therapeutic Oncology and Radiophysics, Haukeland University Hospital, Bergen, Norway.

## Abstract

Anastrozole is the first aromatase inhibitor to show a significant survival advantage over megestrol acetate in post-menopausal women with advanced breast cancer. The rationale for extending the use of aromatase inhibitors to the treatment of early breast cancer is based on the efficacy observed in the advanced setting, combined with good tolerability and a convenient dosing regimen. Furthermore, oestrogen deprivation by ovarian ablation (similar to oestrogen antagonism with tamoxifen) is already established as an effective adjuvant treatment in premenopausal women with modality breast cancer. Anastrozole produces a profound suppression of plasma oestrogen levels which is greater than that obtained with earlier aromatase inhibitors (formestane, aminoglutethimide) or megestrol acetate. This could account for the differences in clinical efficacy seen between anastrozole and megestrol acetate. In terms of benefits over other endocrine agents, anastrozole causes significantly less weight gain than megestrol acetate; it does not have the partial agonist activity of tamoxifen, and is unlikely to lead to tumour stimulation in patients resistant to tamoxifen or to exert proliferative effects on the endometrium. The lack of oestrogen agonist activity, however, may possibly have detrimental effects on bone mineral density and blood lipid profile. Current clinical trials are investigating the efficacy and safety of anastrozole in the early breast cancer setting. The results of these trials will help to determine whether anastrozole has any benefits over tamoxifen, the current treatment of choice in post-menopausal women with early breast cancer.


British Journal of Cancer (1998) **78**(Supplement 4), 12–15
© 1998 Cancer Research Campaign

### Aromatase inhibitors and their future role in post-menopausal women with early breast cancer

**PE Lønning**

Department of Therapeutic Oncology and Radiophysics, Haukeland University Hospital, Bergen, Norway

**Summary** Anastrozole is the first aromatase inhibitor to show a significant survival advantage over megestrol acetate in post-menopausal women with advanced breast cancer. The rationale for extending the use of aromatase inhibitors to the treatment of early breast cancer is based on the efficacy observed in the advanced setting, combined with good tolerability and a convenient dosing regimen. Furthermore, oestrogen deprivation by ovarian ablation (similar to oestrogen antagonism with tamoxifen) is already established as an effective adjuvant treatment in premenopausal women with modality breast cancer. Anastrozole produces a profound suppression of plasma oestrogen levels which is greater than that obtained with earlier aromatase inhibitors (formestane, aminoglutethimide) or megestrol acetate. This could account for the differences in clinical efficacy seen between anastrozole and megestrol acetate. In terms of benefits over other endocrine agents, anastrozole causes significantly less weight gain than megestrol acetate; it does not have the partial agonist activity of tamoxifen, and is unlikely to lead to tumour stimulation in patients resistant to tamoxifen or to exert proliferative effects on the endometrium. The lack of oestrogen agonist activity, however, may possibly have detrimental effects on bone mineral density and blood lipid profile. Current clinical trials are investigating the efficacy and safety of anastrozole in the early breast cancer setting. The results of these trials will help to determine whether anastrozole has any benefits over tamoxifen, the current treatment of choice in post-menopausal women with early breast cancer.

**Keywords:** anastrozole; aromatase inhibitors; early breast cancer; adjuvant

The introduction of the new generation of non-steroidal aromatase inhibitors, as typified by anastrozole, presents an exciting opportunity in the treatment of breast cancer. These agents provide selective (i.e. they do not interact with other enzymes involved in adrenal steroid synthesis) and potent inhibition of the enzyme aromatase (Yates et al, 1996), with a subsequent profound suppression of plasma oestrogens. These properties are combined with good tolerability and a convenient, once-daily dosing regimen (Buzdar et al, 1996*a*). The new aromatase inhibitors may have improved efficacy compared with the standard, second-line endocrine therapies, megestrol acetate and aminoglutethimide. Anastrozole is the first aromatase inhibitor to show a significant survival advantage over megestrol acetate in post-menopausal women with advanced breast cancer who have failed on prior tamoxifen treatment (Roseman et al, 1997). In an overview analysis of two prospective, randomized, phase III trials (Jonat et al, 1995, 1996; Buzdar et al, 1996*a*; Budzar et al, 1997), anastrozole increased the median survival time from 22.5 months (with megestrol acetate) to 26.7 months ($P$ = 0.02) and the 2-year survival rate from 46.3% to 56.1% at a median follow-up duration of 31 months (Roseman et al, 1997). The finding that adjuvant therapy (ovariectomy or tamoxifen treatment) may 'cure' early breast cancer but not advanced disease (despite objective tumour responses) suggests that even modest differences in the effects of drugs used in advanced disease could translate into a real survival benefit in the adjuvant setting.

Anastrozole is the first endocrine agent since tamoxifen to enter large-scale clinical trials of adjuvant therapy in post-menopausal women. This paper describes the rationale for the use of aromatase inhibitors in the adjuvant setting and gives a brief overview of the clinical trials programme.

*Correspondence to:* PE Lønning, Department of Therapeutic Oncology and Radiophysics, Haukeland University Hospital, N-5021 Bergen, Norway

### RATIONALE FOR ADJUVANT USE OF AROMATASE INHIBITORS

The rationale for extending the use of aromatase inhibitors to the treatment of early breast cancer in post-menopausal women is based on several considerations.

### Mechanism of action

Oestrogen deprivation by ovarian ablation is already established as an effective adjuvant treatment in premenopausal women. The beneficial effect of oestrogen antagonism in the treatment of early breast cancer in post-menopausal women has been clearly demonstrated for tamoxifen, which currently may be regarded as the 'gold standard' in this patient population (Early Breast Cancer Trialists' Collaborative Group, 1992). It is therefore possible that oestrogen deprivation through aromatase inhibition could be advantageous in the early breast cancer setting. Indeed, the new-generation aromatase inhibitors have demonstrated superior efficacy compared to conventional therapy for advanced disease (Roseman et al, 1997).

The new-generation aromatase inhibitors produce profound suppression of oestrogen concentrations. With anastrozole, consistency has been shown between the inhibition of whole-body aromatase activity and the suppression of plasma oestrogen levels down to the limits of detection of the assays used (Table 1) (Geisler et al, 1996*a*). The earlier aromatase inhibitors aminoglutethimide (MacNeill et al, 1992) and formestane (Jones et al, 1992) provided high levels of aromatase inhibition (although less than those of anastrozole), but not suppression of plasma oestrogen levels to a similar extent (Lønning, 1996).

**Table 1** Suppression of aromatase activity and plasma oestrogen levels in 10 patients progressing after tamoxifen treatment during treatment with anastrozole (data from Geisler et al, 1996*a*)

| | Mean suppression (%) | |
|---|---|---|
| | **Anastrozole, 1 mg** | **Anastrozole, 10 mg** |
| Aromatase activity | 96.7 | 98.1 |
| Plasma oestrone level | 86.8 | 86.1 |
| Plasma oestradiol level | 84.0 | 83.5 |
| Plasma oestrone sulphate level | 93.5 | 95.7 |

Data from earlier trials evaluating plasma oestrogen suppression with first- and second-generation aromatase inhibitors may not be directly comparable with contemporary results because of the different radioimmunoassays used. More recent assays, despite showing a somewhat better oestrogen suppression, have shown both aminoglutethimide and formestane to be clearly inferior to anastrozole in terms of hormone suppression (Geisler et al, 1996*b*, 1997). Furthermore, megestrol acetate, which might also function as an oestrogen suppressor, reduced plasma oestrogen levels to a markedly smaller degree than anastrozole (Lundgren et al, 1996). It could be speculated, therefore, that the differences in oestrogen suppression between anastrozole and megestrol acetate account for the differences in clinical efficacy of these two endocrine agents.

### Tolerability

New-generation aromatase inhibitors, such as anastrozole, have good tolerability profiles (Buzdar et al, 1996*a*). This is in marked contrast to the first-generation aromatase inhibitor, aminoglutethimide, which is too toxic for adjuvant use (Coombes et al, 1987). In addition, the progestin, megestrol acetate, is poorly tolerated in the treatment of early disease (Pannuti et al, 1988), a finding consistent with its less favourable tolerability compared with new-generation aromatase inhibitors in the treatment of advanced disease (Buzdar et al, 1996*a*; Dombernowsky et al, 1998). One major disadvantage associated with megestrol acetate is weight gain; however, this occurs in significantly fewer patients with advanced breast cancer treated with anastrozole. In one study, 12% of patients treated with megestrol acetate experienced weight gain of over 10% compared with 4% of patients treated with anastrozole, 10 mg ($P$ = 0.002), and only 2% of patients treated with anastrozole, 1 mg ($P$ = 0.0001) (Buzdar et al, 1996*a*). Moreover, patients who receive megestrol acetate continue to gain weight over time (Figure 1). The improved tolerability of anastrozole compared with megestrol acetate has been confirmed over a longer exposure time of 12 months in post-menopausal women with advanced breast cancer (Buzdar et al, 1996*b*). This is important in the adjuvant setting where the need for long-term treatment has been established with tamoxifen, which is significantly more effective when given for 5 years compared with 2 years (Current Trials Working Party of the Cancer Research Campaign Breast Cancer Trials Group, 1996; Fisher et al, 1996; Swedish Breast Cancer Cooperative Group, 1996).

### Adverse events

Based on its mechanism of action, anastrozole would not be expected to exhibit the partial agonist activity associated with anti-oestrogens, such as tamoxifen (Yates et al, 1996). Although anastrozole's

**Figure 1** Weight gain over time in post-menopausal patients with advanced breast cancer treated with anastrozole, 1 mg or 10 mg once daily, or megestrol acetate, 40 mg four times daily. s.e.m., standard error mean. Reproduced with permission from Buzdar et al, 1996*a*

efficacy may be equivalent to that of tamoxifen in the adjuvant setting, the aromatase inhibitor is unlikely to lead to tumour stimulation in tamoxifen-resistant individuals. Similarly, it is unlikely that anastrozole has proliferative effects on the endometrium. Conversely, however, it is also important to bear in mind the possible detrimental effect this lack of oestrogen agonist activity may have on bone mineral density and blood lipid profile. These possible effects of anastrozole in the adjuvant setting are being investigated in subprotocols of the current clinical trials programme.

### CURRENT CLINICAL TRIALS OF ANASTROZOLE

The future role of new-generation aromatase inhibitors in post-menopausal early breast cancer will ultimately be determined by the outcome of a programme of prospective clinical trials. Other novel aromatase inhibitors, such as letrozole and exemestane, are also to be evaluated in the adjuvant setting. The anastrozole trials are designed to compare the aromatase inhibitor as monotherapy with tamoxifen, in combination with tamoxifen and in sequential treatment with tamoxifen. Evidence from experiments in the rat using a chemically induced tumour model suggests that treatment with tamoxifen plus an aromatase inhibitor may be superior to tamoxifen therapy alone (Tominaga et al, 1990; Zaccheo et al, 1993). It may also be possible to take advantage of anastrozole's lack of cross-resistance with other endocrine agents, seen during the treatment of advanced disease, so that sequential therapy can be extended into the adjuvant setting.

### Arimidex, Tamoxifen Alone or in Combination (ATAC) trial

The ATAC trial has been designed to compare 5 years of tamoxifen (Nolvadex) alone with anastrozole (Arimidex) monotherapy and with tamoxifen plus anastrozole as adjuvant treatment in post-menopausal women with early breast cancer (Figure 2) (Baum et al, 1998). The main assessments are time to breast cancer recurrence, overall survival and tolerability. It will be interesting to see whether the superiority of the aromatase inhibitor–tamoxifen combination over tamoxifen alone in the rat tumour model translates into the clinical setting (Zaccheo et al, 1993; Tominaga et al, 1990).

Although clinical trials of a number of aromatase inhibitors are in progress, only the ATAC study assesses the combination of an

© *Cancer Research Campaign 1998*

aromatase inhibitor (anastrozole) plus tamoxifen in one of the treatment arms. Tamoxifen levels were found to be reduced when the agent was administered in combination with the earlier aromatase inhibitor, aminoglutethimide (Lien et al, 1990). Data are now available showing that tamoxifen levels are unaffected when it is co-administered with anastrozole (Dowsett et al, 1998). As a result, the two endocrine therapies can be included in combination without compromising the efficacy of tamoxifen.

### Arimidex–Nolvadex (ARNO) trial

A second anastrozole study, the ARNO trial conducted by the German Breast Cancer Group, involves a sequential treatment option. After 2 years of adjuvant tamoxifen therapy, patients will be randomized to receive either anastrozole for 3 years or tamoxifen for a further 3 years (Figure 3). The primary assessment criteria in this study are overall survival, relapse-free survival, tolerability and quality of life.

The objective of this trial is to evaluate whether the introduction of a new drug after 2 years of tamoxifen therapy can prevent the development of tamoxifen-stimulated tumour progression. In addition, the benefit of anastrozole in the adjuvant setting will be assessed, taking advantage of the demonstrated lack of cross-resistance between these drugs in advanced disease. Some patients who may be about to relapse on tamoxifen may benefit from a treatment that is not cross-resistant with this therapy.

**Figure 2** Protocol for the ATAC trial. Doses given are daily doses

**Figure 3** Protocol for the ARNO trial

### CONCLUSIONS

The new potent aromatase inhibitors, such as anastrozole, have proven benefits in the treatment of advanced breast cancer. It is hoped that the current trials of adjuvant treatment with anastrozole will confirm the benefit of extending the use of these agents to the treatment of early breast cancer in terms of efficacy, tolerability and patient acceptance. This will help to establish their place in the treatment of early breast cancer in post-menopausal women.

### REFERENCES

Baum M and Houghton J (1998) Arimidex, tamoxifen alone or in combination (ATAC) adjuvant trial in post-menopausal breast cancer (abstract P99). *Eur J Cancer* **34**(suppl. 1): S39

Buzdar A, Jonat W and Howell A (1996*a*) Anastrozole, a potent and selective aromatase inhibitor, versus megestrol acetate in postmenopausal women with advanced breast cancer: results of overview analysis of two phase III trials. *J Clin Oncol* **14**: 2000–2011

Buzdar AU, Howell A, Jones S, Blomqvist C, Vogel C, Eirman W, Wolters J, Mauriac L, Eisenberg P, Jonat W, Plourde P and Azab M (1996*b*) Prolonged exposure confirms a favorable tolerability profile of Arimidex (anastrozole) as compared to megestrol acetate (MA) from two large randomised trials in postmenopausal women with advanced breast cancer (ABC) (abstract 100). *Proc Am Soc Clin Oncol* **15**: 109

Buzdar AU, Jones SE, Vogel CL, Wolter J, Plourde P and Webster A (1997) A phase III trial comparing anastrozole (1 and 10 milligrams), a potent and selective aromatase inhibitor, with megestrol acetate in postmenopausal women with advanced breast carcinoma. *Cancer* **79**: 730–739

Coombes RC, Powles TJ, Easton D, Chilvers C, Ford HT, Smith IE, McKinna A, White H, Bradbeer J, Yarnold J, Nash A, Bettelheim R, Dowsett M and Gazet JC (1987) Adjuvant aminoglutethimide therapy for postmenopausal patients with primary breast cancer. *Cancer Res* **47**: 2496–2499

Current Trials Working Party of the Cancer Research Campaign Breast Cancer Trials Group (1996) Preliminary results from the Cancer Research Campaign trial evaluating tamoxifen duration in women aged 50 years or older with breast cancer. *J Natl Cancer Inst* **88**: 1834–1839

Dombernowsky P, Smith I, Falkson G, et al (1998) Letrozole, a new oral aromatase inhibitor for advanced breast cancer: double-blind randomized trial showing a dose effect and improved efficacy and tolerability compared with megestrol acetate. *J Clin Oncol* **16**: 453–461

Dowsett M, Tobias JS, Howell A, Blackman GM, Welch H, King N, Ponzone R, von Euler M and Baum M (1998) The effect of anastrozole on the pharmacokinetics of tamoxifen in postmenopausal women with early breast cancer. *Br J Cancer* (in press)

Early Breast Cancer Trialists' Collaborative Group (1992) Systemic treatment of early breast cancer by hormonal, cytotoxic, or immune therapy. *Lancet* **339**: 1–15, 71–85

Fisher B, Dignam J, Bryant J, et al (1996) Five versus more than five years of tamoxifen therapy for breast cancer patients with negative lymph nodes and estrogen receptor-positive tumors. *J Natl Cancer Inst* **88**: 1529–1542

Geisler J, King N, Dowsett M, Ottestad L, Lundgren S, Walton P, Kormeset PO and Lønning PE (1996*a*) Influence of anastrozole (Arimidex), a selective, non-steroidal aromatase inhibitor, on in vivo aromatisation and plasma oestrogen levels in post-menopausal women with breast cancer. *Br J Cancer* **74**: 1286–1291

Geisler J, Johannessen DC, Anker G and Lønning PE (1996*b*) Treatment with formestane alone and in combination with aminoglutethimide in heavily pretreated breast cancer patients: clinical and endocrine effects. *Eur J Cancer* **32A**: 789–792

Geisler J, Lien EA, Ekse D and Lønning PE (1997) Influence of aminoglutethimide on plasma levels of estrone sulphate and dehydroepiandrosterone sulphate in postmenopausal breast cancer patients. *J Steroid Biochem Mol Biol* **63**: 53–58

Jonat W, Howell A, Blomqvist C, et al (1996) A randomised trial comparing two doses of the new selective aromatase inhibitor anastrozole (Arimidex) with megestrol acetate in postmenopausal patients with advanced breast cancer. *Eur J Cancer* **32A**: 404–421

Jones S, MacNeill FA, Jacobs S, Lønning PE, Dowsett M and Powles TJ (1992) The influence of intramuscular 4-hydroxyandrostenedione on peripheral aromatisation in breast cancer patients. *Eur J Cancer* **28A**: 1712–1716

© Cancer Research Campaign 1998

Lien EA, Anker G, Lønning PE, Solheim E and Ueland PM (1990) Decreased serum concentrations of tamoxifen and its metabolites induced by aminoglutethimide. *Cancer Res* **50**: 5851–5857

Lønning PE (1996) Pharmacology of new aromatase inhibitors. *Breast* **5**: 202–208

Lundgren S, Helle SI and Lønning PE (1996) Profound suppression of plasma estrogens by megestrol acetate in postmenopausal breast cancer patients. *Clin Cancer Res* **2**: 1515–1521

MacNeill FA, Jones AL, Jacobs S, Lønning PE, Powles TJ and Dowsett M (1992) The influence of aminoglutethimide and its analogue rogletimide on peripheral aromatisation in breast cancer. *Br J Cancer* **66**: 692–697

Pannuti F, Martoni A, Cilenti G, Camaggi CM and Fruet F (1988) Adjuvant therapy for operable breast cancer with medroxyprogesterone acetate alone in postmenopausal patients or in combination with CMF in premenopausal patients. *Eur J Cancer Clin Oncol* **24**: 423–429

Roseman BJ, Buzdar AU and Singletary SE (1997) Use of aromatase inhibitors in postmenopausal women with advanced breast cancer. *J Surg Oncol* **66**: 215–220

Swedish Breast Cancer Cooperative Group (1996) Randomized trial of two versus five years of adjuvant tamoxifen for postmenopausal early stage breast cancer. *J Natl Cancer Inst* **88**: 1543–1549

Tominaga T, Yoshida Y, Shimozuma K, Hayashi K and Kosaki G (1990) Effect of CGS 16949 A plus tamoxifen on induced mammary tumours in rats. *Eur J Cancer* **26**: 600–603

Yates RA, Dowsett M, Fisher GV, Selen A and Wyld PJ (1996) Arimidex (ZD1033): a selective, potent inhibitor of aromatase in post-menopausal female volunteers. *Br J Cancer* **73**: 543–548

Zaccheo T, Giudici D and Di Salle E (1993) Inhibitory effect of combined treatment with the aromatase inhibitor exemestane and tamoxifen on DMBA-induced mammary tumors in rats. *J Steroid Biochem Mol Biol* **44**: 677–680

© Cancer Research Campaign 1998